# Ureteral calculus complicated by bladder malakoplakia: A case report

**DOI:** 10.1097/MD.0000000000042926

**Published:** 2025-06-20

**Authors:** Huajian Ye, Liming Yu, Yaokang Chen

**Affiliations:** a Department of Urology, Shaoxing Second Hospital, Shaoxing, Zhejiang Province, People’s Republic of China.

**Keywords:** bladder malakoplakia, case report, Michaelis-Gutmann bodies, ureteral calculus

## Abstract

**Rationale:**

Bladder malakoplakia is a rare granulomatous inflammatory condition that mimics bladder tumors clinically and radiologically. This case is reported to highlight diagnostic challenges and prevent unnecessary radical surgery, emphasizing the critical role of histopathology in identifying this underrecognized entity.

**Patient concerns:**

A female patient presented with recurrent urinary frequency persisting for >1 year. No hematuria, dysuria, or flank pain was reported.

**Diagnoses:**

Diagnoses included bladder tumors, right renal calculus and upper ureteral calculus with hydronephrosis, renal cortical thinning, and hypertension. The postoperative pathology confirmed bladder malakoplakia. Histopathology identified characteristic Michaelis–Gutmann bodies.

**Interventions:**

Transurethral resection of bladder tumor and laparoscopic ureterolithotomy. No adjuvant immunotherapy or long-term antibiotics administered postoperatively.

**Outcomes:**

Successful resection of bladder lesion. Resolution of lower urinary tract symptoms at 3-month follow-up. No recurrence on follow-up cystoscopy.

**Lessons:**

Malakoplakia should be considered in the differential diagnosis of bladder masses, particularly when unresponsive to standard therapies. Histopathological identification of Michaelis–Gutmann bodies remains the diagnostic gold standard. Conservative resection (transurethral resection of bladder tumor) provides both diagnostic confirmation and therapeutic efficacy. Associated urinary tract abnormalities (e.g., obstructive calculi) may contribute to pathogenesis and require concurrent management.

## 1. Introduction

### 1.1. Specific case presentation

Patient information: A female patient was admitted to the hospital due to recurrent urinary frequency persisting for over a year. She initially experienced increased urinary frequency without a clear cause, occurring every 10 to 20 minutes during peak times and up to 5 to 6 times nightly. The patient denied dysuria, hematuria, difficulty urinating, reduced urine stream, fever, abdominal pain, bloating, vomiting, loin pain, oliguria, or edema. B-scan ultrasonography conducted on June 22, 2022, revealed thinning of the right renal cortex, right renal calculi, right upper ureteral calculi with dilation of the right renal pelvis, and a hypoechoic mass within the bladder. No treatment was initiated at that time. On January 16, 2023, computed tomography demonstrated a significant intravesical mass, upper ureteral calculi with marked dilation of the right renal pelvis, and a small cyst in the left kidney. Hospitalization and surgical intervention were recommended. Consequently, the patient presented to the outpatient department and was advised for surgical intervention, subsequently admitted with a provisional diagnosis of “bladder tumor, right ureteral calculus.”

**Figure 1.**
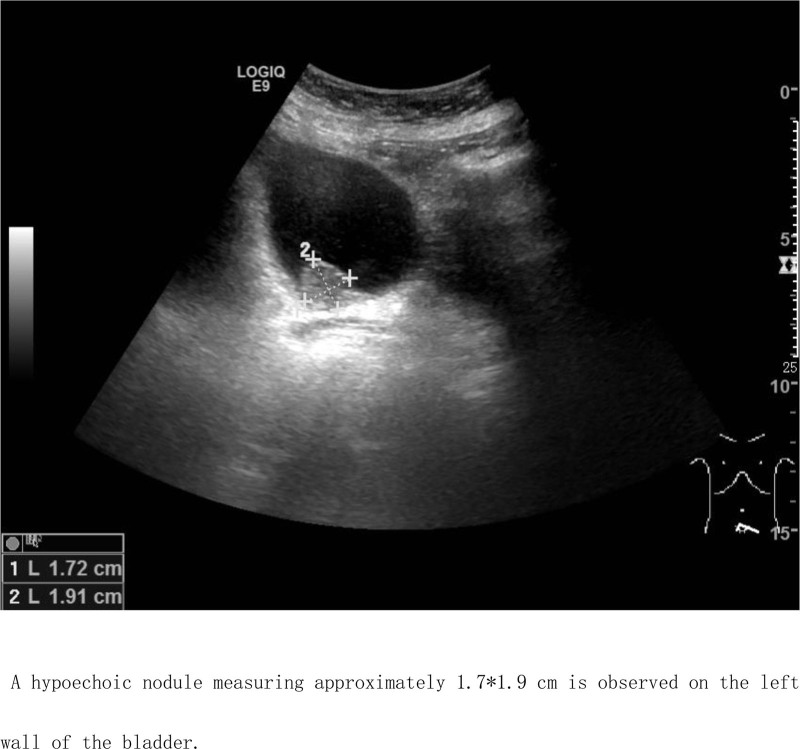
A hypoechoic nodule measuring approximately 1.7 × 1.9 cm is observed on the left wall of the bladder.

**Figure 2.**
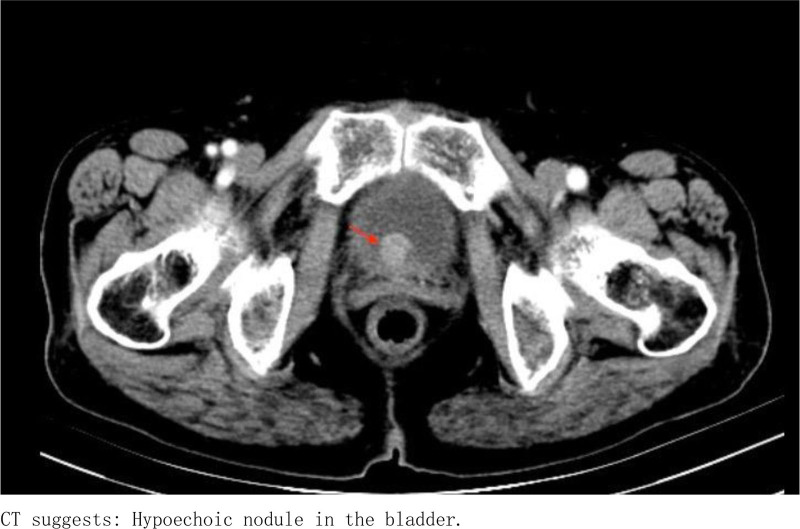
CT suggests: hypoechoic nodule in the bladder. CT = computed tomography.

**Figure 3.**
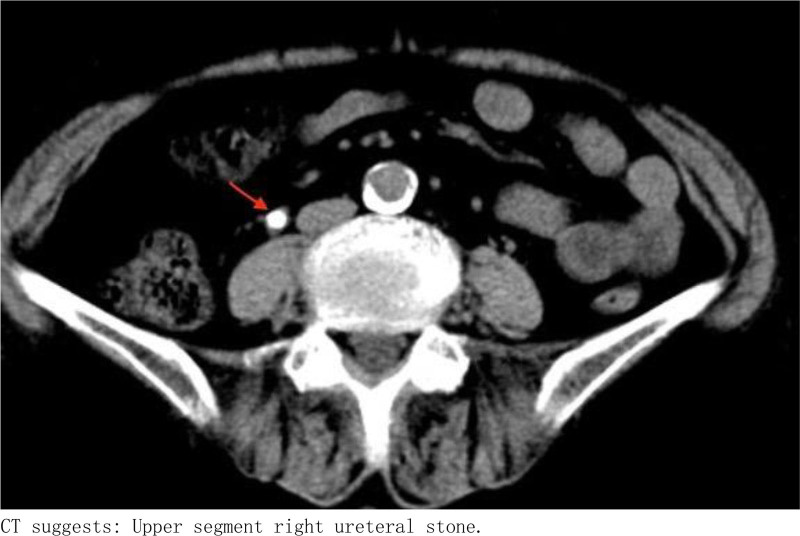
CT suggests: upper segment right ureteral stone. CT = computed tomography.

**Figure 4.**
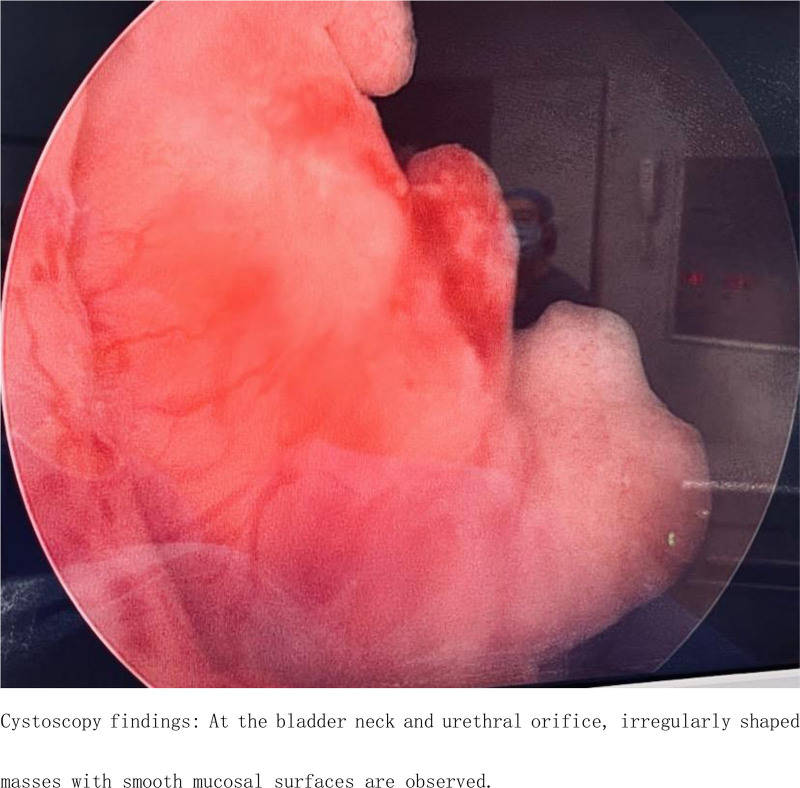
Cystoscopy findings: At the bladder neck and urethral orifice, irregularly shaped masses with smooth mucosal surfaces are observed.

Ancillary investigations: B-scan ultrasonography conducted on June 22, 2022, demonstrated thinning of the right renal cortex, presence of right renal calculi, right upper ureteral calculi with associated right renal pelvis dilation, and a hypoechoic mass within the bladder (Fig. 1). Computed tomography on January 16, 2023, revealed a significant intravesical mass, upper ureteral calculi accompanied by pronounced right renal pelvis dilation, and a small cyst in the left kidney (Figs. 2 and 3). On January 19, 2023, cystoscopy in our hospital revealed a smooth-surfaced, irregularly shaped mass located at the bladder neck and urethral opening, measuring approximately 2.0 × 1.5 cm (Fig. 4).

Past medical history: The patient has a 20-year history of hypertension, controlled with a regular regimen of amlodipine besylate, losartan, and hydrochlorothiazide, maintaining well-controlled blood pressure. Surgical history includes internal fixation surgery for fractures of the left proximal humerus and medial malleolus.

Personal and family history: The patient reports no significant personal medical history, and there is no family history of similar illnesses.

**Figure 5.**
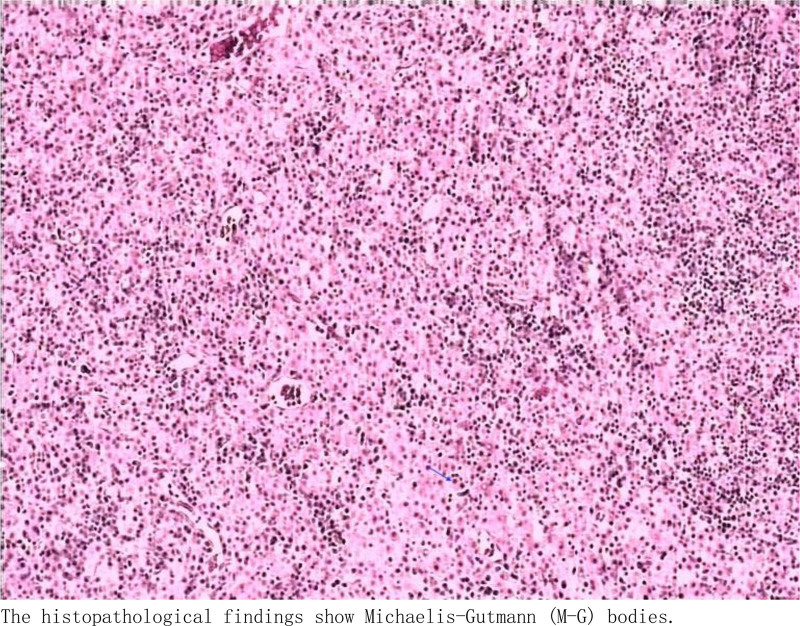
The histopathological findings show Michaelis–Gutmann bodies.

Treatment process: The patient underwent transurethral bladder tumor resection and laparoscopic ureterolithotomy under general anesthesia. Postoperative pathology confirmed the presence of bladder malakoplakia (Fig. 5).

## 2. Discussion

Bladder malakoplakia, also known as von Hansemann disease, was 1st reported by Michaelis and Gutmann in 1902^[1]^ It is an extremely rare chronic infection-related granulomatous disease. Between 1986 and 2021, only 49 worldwide reports on malakoplakia in the bladder were documented.^[2]^ This disease can affect various parts of the body, but it most commonly affects the urinary system, with bladder malakoplakia being the most prevalent, accounting for 40%. The kidneys (29%), testes (12%), ureters (11%), prostate (10%), and urethra (3%) are the next most commonly affected parts.^[3]^ Additionally, it may occur in other parts including the gastrointestinal tract, pancreas, liver, lymph nodes, adrenal glands, vagina, and brain. Malakoplakia in the bladder can affect both men and women, but it is more prevalent in females, particularly in elderly women, with a male-to-female ratio of approximately 1:4. However, there are also reports of its occurrence in children and adolescents.^[4,5]^

The etiology of malakoplakia is currently under research and requires further clarification. Mainstream views currently include only 3 types: bacterial infection, immune response defects, and abnormal macrophage function. The 1st type is bacterial infection, predominantly *Escherichia coli* infections. Prolonged and recurrent infections lead to repeated inflammatory reactions in the bladder mucosa, stimulating granulation tissue hyperplasia.^[6]^ The 2nd type involves chronic diseases or immune response defects, commonly observed in patients requiring immunosuppressive therapy post-organ transplantation, and can also occur in individuals with human immunodeficiency virus infection, pulmonary tuberculosis, malignant tumors, diabetes, and other conditions^[7]^ The 3rd type concerns abnormal macrophage phagocytic digestion function. Macrophages normally extend pseudopods to engulf bacteria, but defects in macrophage lysosomes prevent complete bacterial destruction, resulting in bacterial precipitation within macrophages, combined with iron and calcium deposition, forming specific Michaelis–Gutmann bodies.^[8]^

Due to the low specificity of clinical manifestations and imaging examinations for bladder malakoplakia, distinguishing it from bladder tumors is challenging, necessitating reliance on pathology for diagnosis. Microscopically, typical Michaelis–Gutmannn bodies are frequently observed, appearing as round or oval structures with clear borders. Tissue sections often exhibit clusters of cells along with variable-sized infiltrations of lymphocytes and plasma cells, characterized by cytoplasm rich in eosinophilic granules. Within cells, concentric layers of inclusion bodies resembling target rings or owl eyes are often visible.

Due to the rarity of this disease, there are currently no multicenter randomized controlled treatment case studies reported, and treatment mainly focuses on maintaining urine sterility. Conventional anti-infective therapy, including compound sulfamethoxazole, quinolones,^[9]^ and others, often effectively controls the condition according to literature. However, if conventional drug treatment proves ineffective as the disease progresses, surgical intervention remains necessary.

## 3. Conclusion

The symptoms of bladder malakoplakia are nonspecific, making diagnosis challenging and often resulting in misdiagnosis. Diagnosis relies on histopathological examination. Conventional oral antibiotics can treat this disease, and if treatment fails, transurethral resection of bladder tumor combined with internal medicine treatment can achieve better therapeutic outcomes.

## Author contributions

**Investigation:** Liming Yu, Yaokang Chen.

**Writing** – **original draft:** Huajian Ye.

**Writing** – **review & editing:** Huajian Ye.
